# The Possible Role of Neurobeachin in Extinction of Contextual Fear Memory

**DOI:** 10.1038/s41598-018-30589-1

**Published:** 2018-09-13

**Authors:** Boyoung Lee, Eunyoung Bang, Won Suk Yang, Afshin Paydar, Go Eun Ha, Sujin Kim, Jong-Hyun Kim, Taesup Cho, Seung Eun Lee, Sukchan Lee, Myoung-Goo Kang, Eunji Cheong, Key-Sun Kim, Cheolju Lee, Myeong-Hee Yu, Hee-Sup Shin

**Affiliations:** 1 Center for Cognition and Sociality, Institute for Basic Science, Daejeon, 34047 Republic of Korea; 2 Basic Science, IBS School, University of Science and Technology, Daejeon, 34113 Republic of Korea; 3 Brain Science Institute, Korea Institute of Science and Technology, Seoul, 02792 Republic of Korea; 4 Laboratory of Cell Death and Human Diseases, Department of Life Sciences, School of Life Sciences, Korea University, Seoul, 02841 Republic of Korea; 5 Department of Biotechnology, College of Life Science and Biotechnology, Yonsei University, Seoul, 03722 Republic of Korea; 6 Center for Theragnosis, Korea Institute of Science and Technology, Seoul, 02792 Republic of Korea; 7 Department of Converging Science and Technology, KHU-KIST, Seoul, 02447 Republic of Korea

## Abstract

Established fear memory becomes vulnerable to disruption after memory retrieval and extinction; this labile state is critical for inhibiting the return of fear memory. However, the labile state has a very narrow time window after retrieval, and underlying molecular mechanisms are not well known. To that end, we isolated the hippocampus immediately after fear memory retrieval and performed proteomics. We identified Neurobeachin (NBEA), an autism-related regulator of synaptic protein trafficking, to be upregulated after contextual fear memory retrieval. NBEA protein expression was rapid and transient after fear memory retrieval at the synapse. *Nbea* mRNA was enriched at the synapses, and the rapid induction of NBEA expression was blocked by inhibition of the mammalian target of rapamycin (mTOR)-dependent signaling pathway. Mice with cornu ammonis 1 (CA1)-specific *Nbea* shRNA knockdown showed normal fear acquisition and contextual fear memory but impaired extinction, suggesting an important role of *Nbea* in fear memory extinction processes. Consistently, *Nbea* heterozygotes showed normal fear acquisition and fear memory recall but showed impairment in extinction. Our data suggest that NBEA is necessary either for induction of memory lability or for the physiological process of memory extinction.

## Introduction

Post-traumatic stress disorder (PTSD) is a neuropsychiatric disorder developed from exposure to severe traumatic events^1^. Contextual fear conditioning is a well-known protocol used to understand context-dependent fear memory consolidation, reconsolidation and extinction processes in which the hippocampus is the core brain region involved^2^. Extinction therapy has been widely used for the treatment of PTSD; however, not all populations of patients experience the beneficial effects of these therapies, and effective pharmacological interventions for PTSD patients are limited^3^. Fear memory retrieval renders the original memory labile, allowing a short time window for amnesic intervention. Then, reconsolidation restores the stability of the memory^4^. However, the labile state has a very narrow time window after retrieval, and the underlying molecular mechanisms are not well known.

*De novo* protein synthesis has been shown to be involved in extinction^5^ and memory lability to trigger instability of the original memory^6,7^. A memory-labile state is rapidly established after retrieval and has a very narrow time window^8^. Recently, the mammalian target of rapamycin (mTOR) has been widely accepted to be involved in local *de novo* protein synthesis, and its target proteins have been shown to play diverse roles in synaptic plasticity and activity-dependent synaptic transmission. The mTOR signaling pathway has been suggested to be involved in rapid modulation of memory susceptibility^9,10^. However, which molecules are rapidly expressed after fear memory retrieval and the roles of these newly synthesized proteins in memory susceptibility and extinction have not been studied.

Neurobeachin (NBEA) is a large, cytosolic multidomain protein selectively expressed in neurons and endocrine cells^11^. NBEA has been suggested to regulate postsynaptic neurotransmitter and ionotropic receptor trafficking to the cell surface^12,13^ and act as a negative regulator of regulated secretion^14^. NBEA also regulates synaptic architecture through actin cytoskeleton remodeling^15^. In zebrafish, NBEA is essential for the maintenance of extensive dendritic branching in mature neurons^16^. Further, NBEA was recently reported to regulate Notch-mediated gene transcription^17^. NBEA also directly interacts with protein kinase A (PKA) and regulates PKA-mediated phosphorylation^11^. Because *Nbea* is a candidate gene for autism spectrum disorder (ASD), *Nbea* heterozygous mice were generated using the gene-trap method, and 24-month-old female mice were phenotyped^18,19^. However, there are insufficient studies to identify the link between the molecular role of NBEA and the resulting behavioral phenotypes, especially fear memory processes.

Here, we performed unbiased proteomics to isolate the molecules involved in fear memory retrieval and found that NBEA was upregulated in the dorsal hippocampus. *Nbea* mRNA was enriched at the synapse, in which its expression was rapidly and transiently expressed immediately after retrieval. Suppression of NBEA expression by knockdown and knockout impaired extinction, suggesting that NBEA is necessary for either the induction of memory lability or the physiological process of memory extinction.

## Results

### Isolation of NBEA from the proteomic analysis after contextual fear memory retrieval

**Figure 1:**
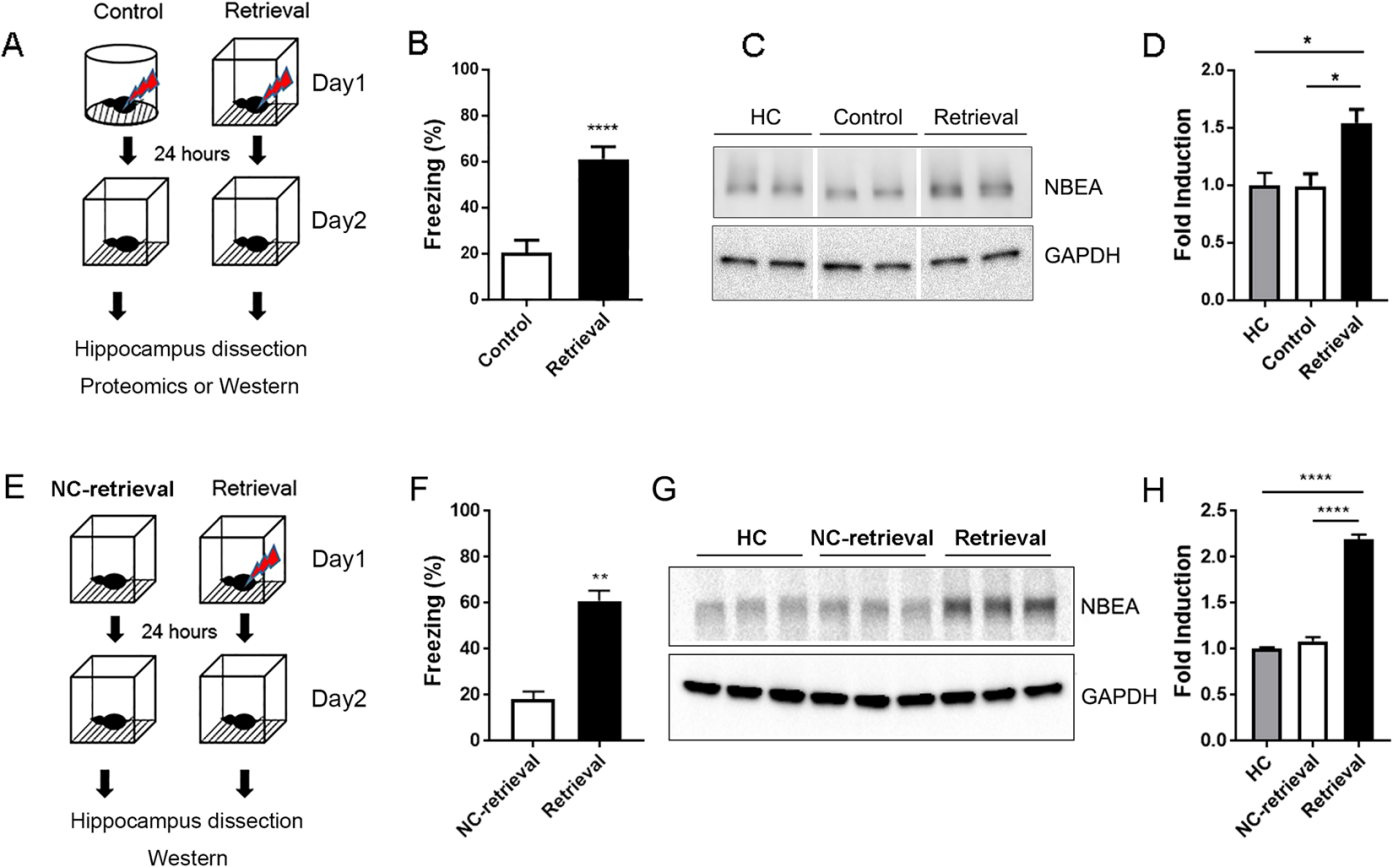
Proteomic analysis identified NBEA as one of the candidate molecules regulated by contextual fear retrieval in the hippocampus. (**A**) Schematic representation of the experimental protocol. Mice in the control group were fear conditioned with a shock in context A and underwent a retrieval test in context B. Mice in the retrieval group were fear conditioned with a shock in context B and underwent a retrieval test in the same context (context B). (**B)** Freezing level (%) during 5-min retrieval test. Freezing level in the retrieval group (61.19 ± 5.283, n = 10) was significantly higher than that in the control group (20.48 ± 5.381, n = 10), ***p < 0.0001, t-test, two-tailed. (**C**) Western blot image of NBEA expression in the control and retrieval group. GAPDH was used for the loading control. (**D**) Intensity measurement of the Western blot. NBEA expression was significantly higher in the retrieval group (1.541 ± 0.1187, n = 3) than in the control group (0.9913 ± 0.1091, n = 3), *p = 0.0327. NBEA expression was significantly higher in the retrieval group (1.541 ± 0.1187, n = 3) than in the home cage (HC) group (1 ± 0.1077, n = 3), *p = 0.0307, one-way ANOVA, Tukey’s multiple comparisons post hoc test. (**E**) Schematic representation of the experimental protocol. Mice in the NC (neutral context) retrieval group were handled as the same as the retrieval group with no shock of Day 1. (**F**) Freezing level in the retrieval group (60.89 ± 4.415, n = 3) was significantly higher than that in the NC (neutral context)-retrieval group (18.31 ± 3.179, n = 3), **p = 0.0014, t-test, two-tailed. (**G**) Western blot image of NBEA expression in the home cage (HC), NC-retrieval and retrieval groups. GAPDH was used as the loading control. (**H**) Intensity measurements of the Western blots. NBEA expression was significantly higher in the retrieval group (2.193 ± 0.05042, n = 3) than in the HC group (1 ± 0.01841, n = 3), ****p = 0.0001. NBEA expression was significantly higher in the retrieval group (2.193 ± 0.05042, n = 3) than in the NC-retrieval group (1.084 ± 0.04583, n = 3), ****p = 0.0001. There was no difference between the NC-retrieval group (1.084 ± 0.04583, n = 3) and the HC group in NBEA expression, p = 0.3146, one-way ANOVA, Tukey’s multiple comparisons post hoc test.

Memory retrieval renders the consolidated memory labile and that extinction training during the labile state can alter or erase fear memory (Fukushima *et al*.^7^). However, the molecular mechanisms underlying the memory retrieval-mediated process are not well known. To identify candidate molecules involved in fear memory retrieval, we utilized unbiased proteomics analysis. To isolate candidate molecules specific to the retrieval process, both a control and retrieval group were exposed to a foot shock (0.5 mA, 1 s) for the contextual fear conditioning. The next day, mice in the control group were placed in a different context from the conditioned context, and the retrieval group was placed in the same context (Fig. 1A). Importantly, there was a significant difference in the freezing level between groups on the retrieval test (Fig. 1B; t(18) = 5.398, ***p < 0.0001, unpaired t-test, two-tailed). Then, proteins were isolated from the hippocampus 5 min after fear memory retrieval and further processed for proteomic analysis. The peptide mixture from 5 mice of each group (control and retrieval) was labeled with TMT reagents. These labeled peptides were analyzed with LC-MS/MS analysis using an LTQ Orbitrap XL. A summary of the differentially expressed proteins (DEPs) is presented in Table 1. Among the 14 regulated proteins identified in the proteomic analysis, we decided to focus on the role of NBEA in the fear memory process because NBEA is known to regulate synaptic transmission by modulating the trafficking of receptors, including AMPA receptors, at synapses^12,13,20^. Recently, several studies have reported that AMPA receptor trafficking is significant for memory stabilization and destabilization after retrieval^21,22^. We confirmed the increased expression of NBEA after contextual fear retrieval. Since we focused on the rapid induction of protein expression and given the major role of NBEA in trafficking neurotransmitter receptors, we isolated synaptosomes from the dorsal hippocampus to examine the specific role of NBEA at the synapse. Here, NBEA expression at the synapse was significantly increased 5 min after memory retrieval compared to expression in the control group (Fig. 1C,D, Supple Fig. 4; F(2, 6) = 7.916, *p = 0.0208, one-way ANOVA, Tukey’s multiple comparisons post hoc test, HC vs Retrieval, *p = 0.0327, Control vs Retrieval, *p = 0.0307). Home cage samples were used to observe the basal expression of NBEA. Next, we further examined whether the increase in NBEA expression is specific to fear retrieval. To that end, mice were exposed to the same context as the retrieval group without foot shock on day 1 and re-exposed to the same context on day 2, which we described as neutral context retrieval (NC-retrieval) (Fig. 1E). Interestingly, compared to the home-cage group (HC), the NC-retrieval group expressed a similar amount of NBEA, but the retrieval group showed a significant induction in NBEA expression (Fig. 1G,H; F(2, 6) = 267, ****p < 0.0001, one-way ANOVA, Tukey’s multiple comparisons post hoc test, HC vs Retrieval, ****p < 0.0001, NC-retrieval vs Retrieval, ****p < 0.0001), indicating that the rapid increase in NBEA expression was fear-specific. The freezing level confirmed the delivery of foot shocks (0.7 mA, 1 s, 2 shocks) to retrieval group (Fig. 1F; t(4) = 7.826, **p = 0.0014, unpaired t-test, two-tailed).

**Table 1:** Proteins significantly changed by contextual fear retrieval.

IPI	Swiss-Prot	Protein	Description	# unique peptides	Fold change (retrieval/control)
IPI00313151	O35098	DPYSL4	Dihydropyrimidinase-related protein 4	4	0.50
IPI00387430	Q9D6J5	NDUFB8	NADH dehydrogenase [ubiquinone] 1 beta subcomplex subunit 8	2	0.50
IPI00135563	P55066	NCAN	Neurocan core protein precursor	4	0.55
IPI00132460	P61255	RPL26	60S ribosomal protein L26	2	0.59
IPI00154057	Q2TJH4	PCDH1	Protocadherin 1	5	0.61
IPI00420651	Q6R891	PPP1R9B	Neurabin-2	3	0.64
**IPI00471441**	**Q9D0J8**	**PTMS**	**Ptms protein**	**4**	**1.50**
**IPI00115680**	**O08795**	**PRKCSH**	**Glucosidase 2 subunit beta precursor**	**2**	**1.50**
**IPI00460558**	**Q6PH08**	**ERC2**	**ERC protein 2**	**2**	**1.50**
**IPI00652358**	**P50429**	**ARSB**	**Isoform 1 of Arylsulfatase B precursor**	**4**	**1.54**
**IPI00659932**	**Q3UVX5**	**GRM5**	**Metabotropic glutamate receptor 5 precursor**	**2**	**1.56**
**IPI00320831**	**Q9EPN1**	**NBEA**	**Neurobeachin**	**3**	**1.63**
**IPI00403890**	**O88448**	**KLC1**	**kinesin light chain 1 isoform 1D**	**2**	**1.67**
**IPI00420136**	**O70492**	**SNX3**	**Sorting nexin 3**	**4**	**1.69**

Significantly changed proteins are listed. The control group was exposed to an unconditioned stimulus (US) but retrieved memories in a different context than the conditioning context. The retrieval group retrieved memories in the same context as the conditioning context. Downregulated proteins are indicated in regular typeface, and upregulated proteins are indicated in bold typeface.

### Rapid and transient expression of NBEA after fear memory retrieval

**Figure 2:**
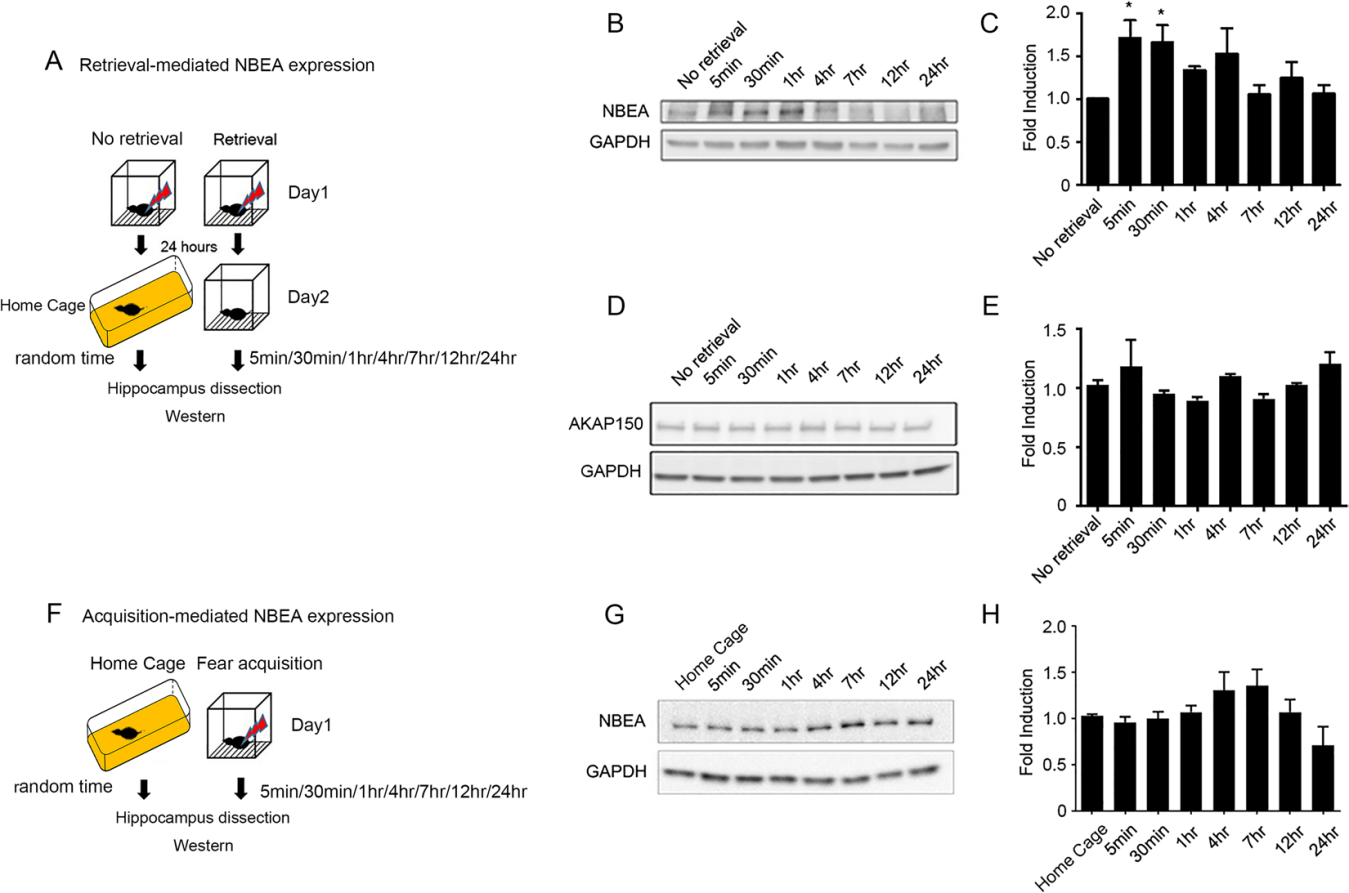
The increase in NBEA expression was rapid and transient after contextual fear retrieval at the synapse in the dorsal hippocampus. (**A**) Schematic representation of the experimental protocol. Mice in the no-retrieval group were handled as the same as the retrieval group on Day 1 but were not exposed to the same context on Day 2. Random time means the no-retrieval group was sampled randomly, but most of the samples were taken between the 1 hr and 4 hr sampling intervals after fear retrieval. (**B**) Representative image of the time course of NBEA expression after retrieval. No-retrieval samples were obtained from mice that were fear conditioned and sacrificed 24 h after conditioning without retrieval. (**C**) Quantification of the time course of NBEA expression after retrieval. There was significant induction in NBEA expression at 5 min and 30 min after contextual fear retrieval. F(7, 39) = 2.793, *p = 0.0187, one-way ANOVA with post hoc Dunnett’s multiple comparisons test: No retrieval (1.005 ± 0.003658, n = 6) vs 5 min after retrieval (1.713 ± 0.2036, n = 5), *p = 0.0328; no retrieval vs 30 min after retrieval (1.661 ± 0.2003, n = 6), *p = 0.0400; no retrieval vs 1 h after retrieval (1.339 ± 0.04178, n = 6), p = 0.5568; no retrieval vs 4 h after retrieval (1.529 ± 0.2942, n = 6), p = 0.1411; no retrieval vs 7 h after retrieval (1.056 ± 0.1076, n = 6), p = 0.9997; no retrieval vs 12 h after retrieval (1.25 ± 0.1824, n = 6), p = 0.8216; no retrieval vs 24 h after retrieval (1.069 ± 0.09385, n = 6), p = 9996. (**D**) Representative image of the time course of AKAP150 expression after retrieval. (**E**) Quantification of the time course of AKAP150 expression after retrieval. There was no significant difference in AKAP150 expression after contextual fear retrieval. F(7, 28) = 2.021, p = 0.0877, one-way ANOVA. (**F**) Schematic representation of the experimental protocol. Random time means the home cage group was sampled randomly, but most of the samples were taken between the 1 hr and 4 hr sampling intervals after fear training. (**G**) Representative image of the time course of NBEA expression after conditioning. Home-cage samples were obtained from mice directly taken from the home cage and sacrificed. (**H**) Quantification of the time course of NBEA expression after fear conditioning. There was no significant difference in NBEA expression after fear conditioning. F (7, 20) = 2.255, p = 0.0728, one-way ANOVA.

Next, we further examined the expression pattern of NBEA at the synapse at different time points after memory retrieval. Interestingly, the immediate induction of NBEA expression 5 min after retrieval lasted up to 4 h and dropped by 6 h after retrieval, correlating very well with the time course of the labile state of fear memory (Fig. 2A,B,C; F(7, 39) = 2.793, *p = 0.0187, one-way ANOVA). Since, NBEA is a well-known AKAP protein, which binds to PKA and regulates PKA signaling pathways, we also examined the expression of another well-studied AKAP protein, AKAP150, to determine whether the dynamic expression of NBEA after retrieval was unique to NBEA. Western blot analysis showed that there were no significant changes in AKAP150 expression after retrieval compared to the expression in the no-retrieval samples (Fig. 2D,E; F(7, 28) = 2.021, p = 0.0877, one-way ANOVA), suggesting that the dynamic expression after retrieval was not shown in one of the major AKAP isoforms present in the hippocampus^23,24^. In this experiment, the no-retrieval group, which was not exposed to any context after fear conditioning, was used as a control to examine NBEA expression 24 h after conditioning but immediately before retrieval. To determine whether this dynamic expression was specific to the retrieval process, we analyzed the time course of NBEA expression after fear conditioning. Interestingly, we did not find any significant changes in NBEA expression after fear conditioning, suggesting that the rapid induction of NBEA expression was specific to the fear retrieval process (Fig. 2F,G,H; F(7, 20) = 2.255, p = 0.0728, one-way ANOVA).

### *De novo* local protein synthesis of NBEA after retrieval

**Figure 3:**
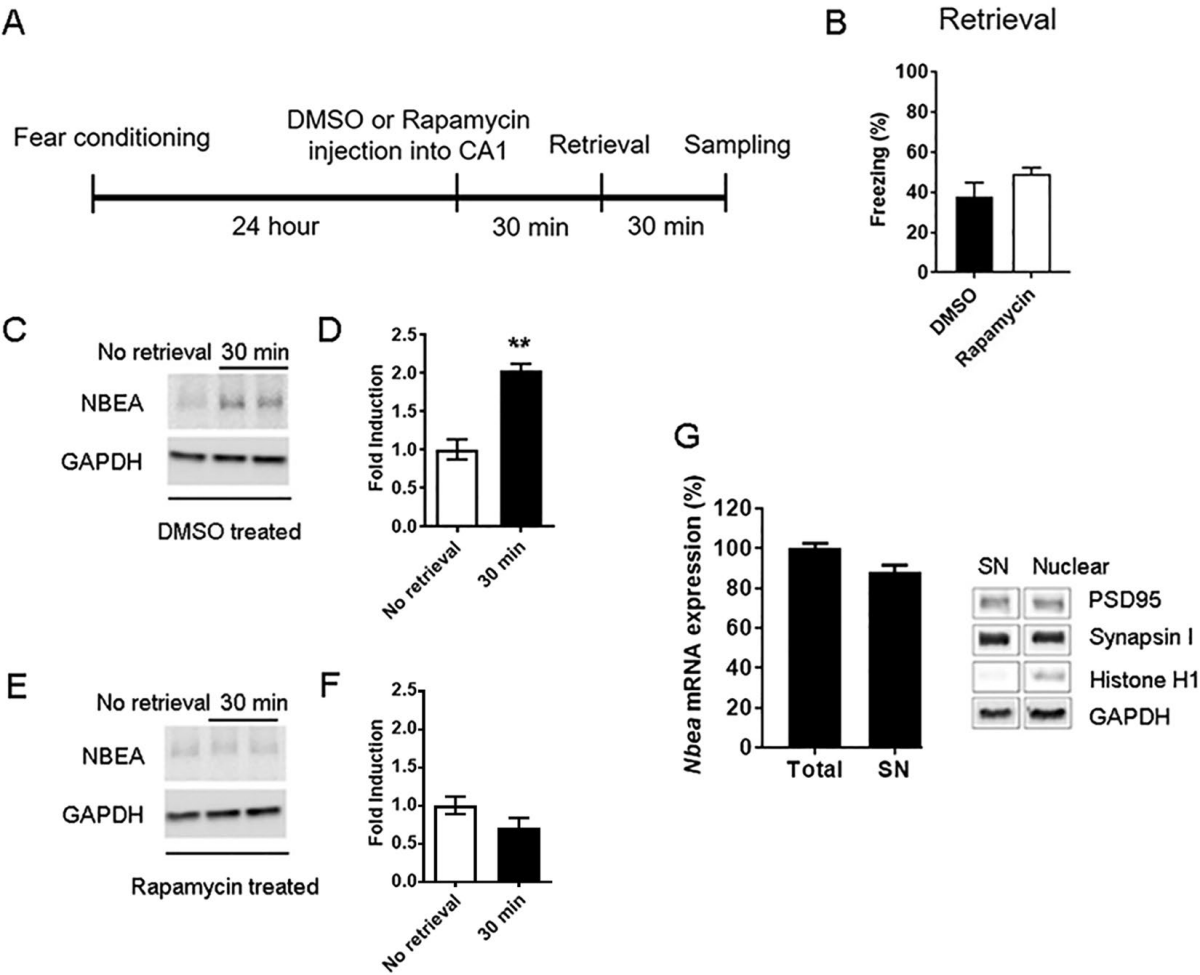
Increased NBEA expression after contextual fear retrieval at the synapse in the dorsal hippocampus was regulated by the mTOR signaling pathway. (**A**) Schematic representation of the experimental protocol. (**B**) Injection of rapamycin (49.03 ± 3.28, n = 4) into the dorsal CA1 hippocampus 30 min before contextual retrieval did not alter the freezing level during retrieval compared to injection of DMSO control (37.95 ± 6.934, n = 4). p = 0.1989, t-test, two-tailed. (**C**) Representative image of NBEA expression with DMSO treatment 30 min before contextual retrieval. (**D**) Quantification of NBEA expression with DMSO treatment. No retrieval (1.004 ± 0.1315, n = 3) vs 30 min after retrieval (2.038 ± 0.0843, n = 3), *p = 0.0027, t-test, two-tailed. (**E**) Representative image of NBEA expression with rapamycin treatment 30 min before contextual retrieval. (**F**) Quantification of NBEA expression with rapamycin treatment. No retrieval (1.005 ± 0.1142, n = 3) vs 30 min after retrieval (0.7149 ± 0.1254, n = 3), p = 0.1625, t-test, two-tailed. (**G**) Left, *Nbea* mRNA expression in total lysates (100.2 ± 2.341, n = 9) and the synaptosome fraction (88 ± 3.487, n = 9). Right, Confirmation of the purity of the synaptosome fraction, showing no nuclear contamination using the nuclear marker, histone H1.

**Figure 4:**
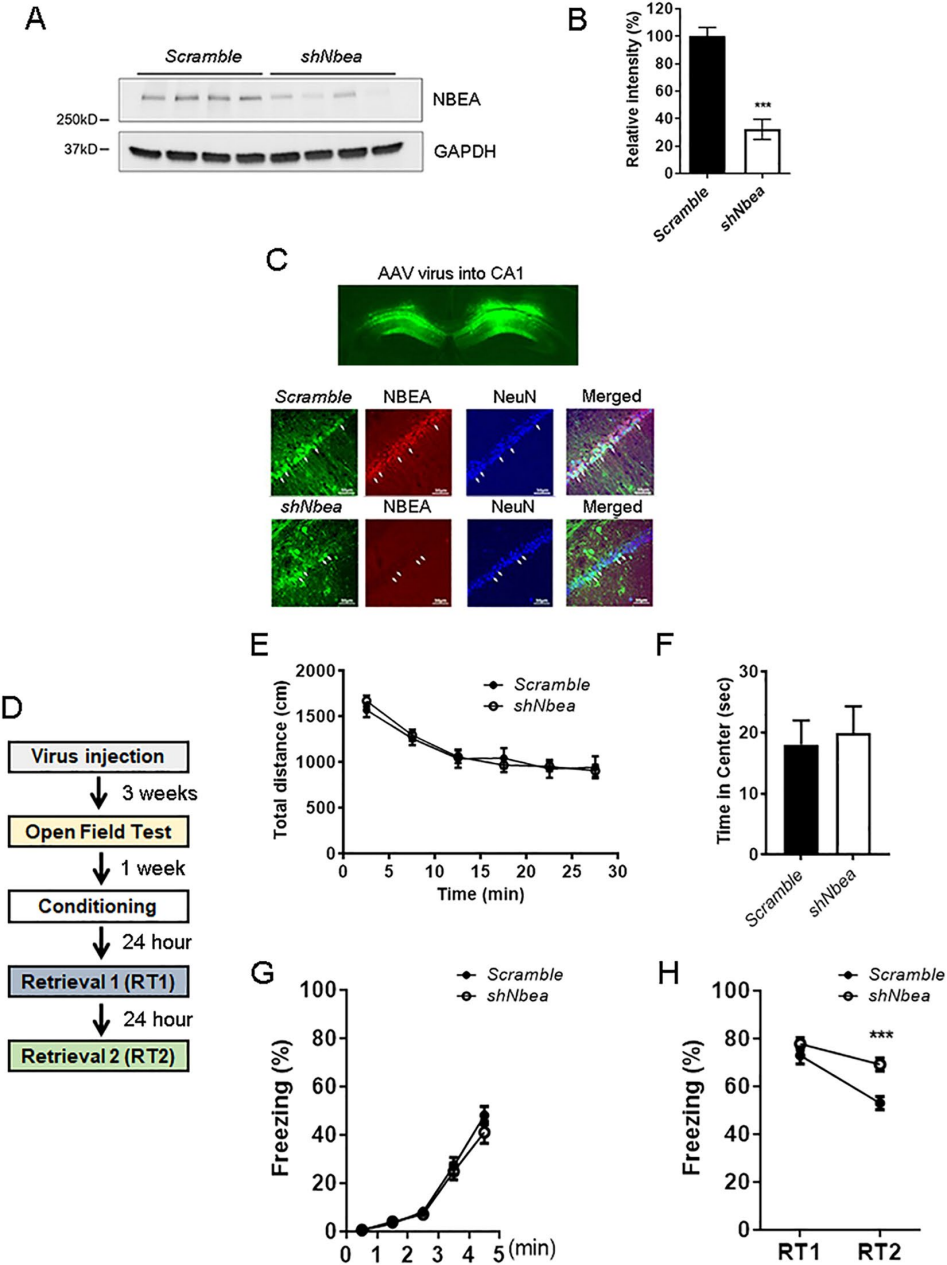
*Nbea* knockdown in the dorsal hippocampal CA1 region resulted in impairments in extinction after contextual fear retrieval. (**A**) Validation of sh*Nbea* efficiency in cultured hippocampal neurons. Left, Western blot image of NBEA expression from AAV-scrambled or AAV-*shNbea-*treated cultured hippocampal neurons. Right, Quantification of NBEA expression. Scrambled (100 ± 6.321, n = 4) vs *shNbea* (32.14 ± 7.286, n = 4), ***p = 0.0004, t-test, two-tailed. (**B**) Upper, Representative image of AAV virus injection into the CA1 of the dorsal hippocampus. Middle, Immunohistochemistry for NBEA and NeuN in AAV-scrambled-injected CA1. Lower, Immunohistochemistry for NBEA and NeuN in AAV-sh*Nbea*-injected CA1. (**C**) Locomotor activity in the open field test. There was no significant difference in the locomotor activity between AAV-scrambled and AAV-*shNbea*-injected mice. F(5, 125) = 0.692, p = 0.6304, two-way ANOVA, post hoc Sidak’s multiple comparison test. Scale bars indicate 50 μm. (**D**) Anxiety-like behavior in the open field test. There was no significant difference in time spent in the center of the open field for the first 5 min of the test between the AAV-scrambled and AAV-*shNbea*-injected mice. Scrambled (18.05 ± 3.96, n = 14) and *shNbea* (19.94 ± 4.404, n = 13), p = 0.7519, t-test, two-tailed. (**E**) Schematic representation of the experimental protocol. (**F**) Fear conditioning. There was no significant difference in the freezing level during fear conditioning between the AAV-scrambled and AAV-*shNbea*-injected mice. F(4, 100) = 0.8908, p = 0.4725, two-way ANOVA, post hoc Sidak’s multiple comparison test. (**G**) Fear retrieval. F(1, 25) = 8.984, **p = 0.0061, two-way ANOVA with post hoc Sidak’s multiple comparisons test. There was no significant difference in the freezing level during retrieval 1 (RT1) between AAV-scrambled and AAV-*shNbea*-injected mice. Scrambled (72.93 ± 3.599, n = 14) and *shNbea* (69.17 ± 2.744, n = 13), p = 0.2945. However, there was a significant difference in the freezing level during retrieval 2 (RT2) between AAV-scrambled and AAV-*shNbea*-injected mice. Scrambled (53 ± 2.733, n = 14) and *shNbea* (77.75 ± 2.603, n = 13), ***p = 0. 0.0003.

The rapid induction of NBEA expression allowed us to investigate the activation of local NBEA protein synthesis through the mTOR signaling pathway, which is known to be involved in rapid local protein synthesis^25,26^. Whether NBEA protein can be locally synthesized and expressed in an activity-dependent manner is unknown. To that end, rapamycin (1 μl of 50 μM in DMSO) or vehicle (DMSO) was administered into the CA1 of dorsal hippocampi 30 min before fear memory retrieval, followed by protein extraction 30 min after retrieval (Fig. 3A). Rapamycin infusion 30 min before retrieval did not change the level of freezing during retrieval (Fig. 3B; t(6) = 1.445, p = 0.1986, unpaired t-test, two-tailed) as shown in other reports^27,28^. However, the rapid induction of NBEA expression shown in the DMSO-infused group (Fig. 3C,D; t(4) = 6.625, *p = 0.0027, unpaired t-test, two-tailed) was completely blocked by rapamycin infusion (Fig. 3E,F; t(4) = 1.710, p = 0.1625, unpaired t-test, two-tailed), suggesting that NBEA is newly and rapidly synthesized at the synapse in response to fear memory retrieval through the mTOR signaling pathway. We also confirmed that NBEA mRNA was enriched in the purified synaptosome (Fig. 3G left), suggesting that NBEA was indeed actively synthesized at the synapse after contextual fear retrieval. The purity of the synaptosomes was validated with the histone H1 antibody to ensure that there was no nuclear contamination (Fig. 3G right and Supple Fig. 4).

### *Nbea* knockdown in the CA1 hippocampus impaired extinction

Since NBEA expression was increased in the hippocampus after contextual fear memory retrieval, we injected AAV encoding a sequence to knockdown NBEA (sh*Nbea*) or a scrambled sequence into the CA1 of the dorsal hippocampus, known to be involved in contextual fear retrieval and extinction, in WT mice. Before we tested the efficiency of the shRNA virus, we analyzed the baseline expression of NBEA in control mouse brains. In the hippocampus, NBEA was expressed in dentate gyrus (DG), CA3 and CA1 pyramidal cells (Supple Fig. S1A). Double staining with parvalbumin showed weak or no expression of NBEA in parvalbumin-positive cells in the thalamic reticular nucleus (TRN; Supple Fig. S1B), cortex (Supple Fig. S1C) and CA1 hippocampus (Supple Fig. S1D). To further confirm that NBEA is more weakly expressed in inhibitory neurons than in pyramidal cells, we examined the expression of NBEA in hippocampal tissue double-labeled with an antibody targeting GABA, an inhibitory neurotransmitter (Supple Fig. S1E), and in tissue from glutamate decarboxylase 67 (GAD67)-GFP transgenic mice to visualize GAD67-positive cells (Supple Fig. S1F). We did not see NBEA expression in those inhibitory neuronal populations; therefore, we concluded that baseline NBEA expression in inhibitory neurons is minimal. The efficiency of sh*Nbea* knockdown (KD) and the scrambled shRNA was confirmed with immunoblotting of lysates from each of the virus-treated *in vitro* primary hippocampal cultures (Fig. 4A,B; t(6) = 7.038, ***p = 0.0004, unpaired t-test, two-tailed). Furthermore, immunohistochemistry data clearly showed a suppression of NBEA expression with sh*Nbea* KD in the CA1 of the dorsal hippocampus but not in the group treated with the scrambled control (Fig. 4C). For the behavior analysis (Fig. 4D), we first examined general locomotor activity and anxiety-like behavior in the open field test. sh*Nbea* KD in the CA1 did not affect locomotion (Fig. 4E; F (5, 150) = 0.2602, p = 0.9341, two-way ANOVA) or anxiety (Fig. 4F; t(25) = 0.3197, p = 0.7519, unpaired t-test, two-tailed). Fear conditioning showed that sh*Nbea* KD in the CA1 did not affect fear acquisition (Fig. 4G; F (4, 100) = 0.8908, p = 0.4725, two-way ANOVA). Retrieval was tested with two sequential retrieval tests (RT1 and RT2) with a 24-h interval. To assess fear memory extinction within the group, we compared the freezing level between RT1 and RT2 of each group. As we observed from the *Nbea*^+/−^ mutant data in Fig. 4D, th esh*Nbea* KD group also showed attenuated extinction (Fig. 4H; sh*Nbea*, RT1-RT2: t(25) = 7.587, ****p < 0.0001, two-way ANOVA, Sidak’s multiple comparisons test) compared to the scrambled control (Fig. 4H; scramble, RT1-RT2: t(25) = 3.148, **p = 0.0084, two-way ANOVA, Sidak’s multiple comparisons test). Interestingly, we did not observe a significant effect of sh*Nbea* KD in RT1, the 24-h fear memory retrieval test performed 24 h after conditioning (Fig. 4H RT1; t(25) = 1.071, p = 0.2945, unpaired t-test, two-tailed), but we observed a significant difference in the behavior between the scrambled control and sh*Nbea* KD group in RT2 (Fig. 4H; F(1, 25) = 8.984, *p = 0.0061, two-way ANOVA, Sidak’s multiple comparisons test, RT1, p = 0.4475, RT2, ***p = 0.0007). These data indicate that the rapid induction of NBEA expression in pyramidal neurons in the CA1 hippocampus may play a role in either the inhibition of memory stability or the facilitation of memory instability after retrieval.

### Neurobeachin heterozygous mice showed impaired extinction

**Figure 5:**
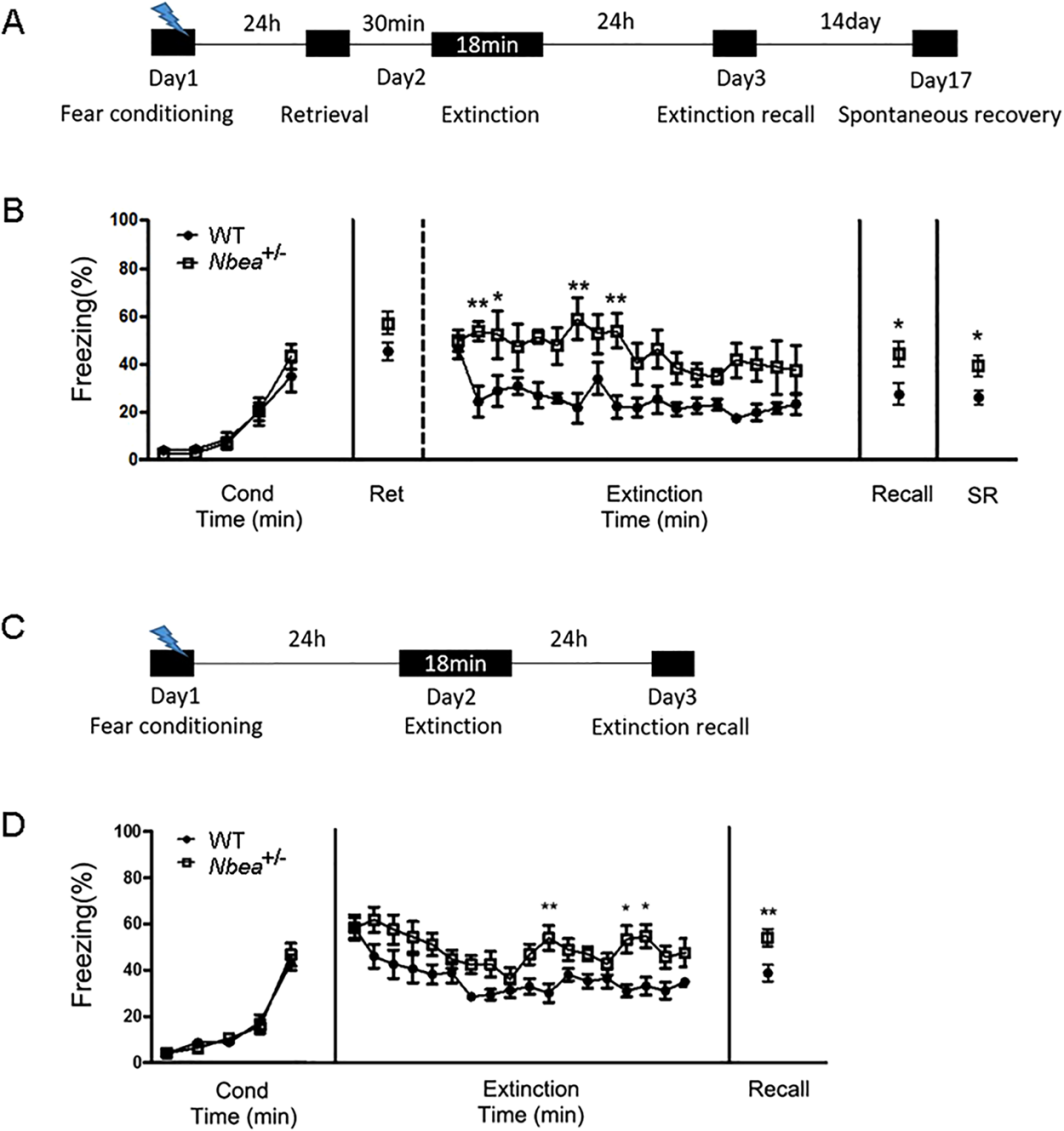
*Nbea*^+/−^ showed impairments in fear extinction. (**A**) Schematic representation of the experimental protocol. (**B**) Fear conditioning: There was no significant difference in freezing level during fear conditioning between WT (n = 7) and *Nbea*+/− (n = 10). Two-way ANOVA, F(4, 60) = 2.19, p = 0.0809. Retrieval: There was no difference in retrieval. WT (47.23 ± 3.587, n = 7) vs *Nbea*+/− (56.68 ± 3.524, n = 10), p = 0.0881, unpaired t-test, two-tailed. Extinction: There was a group difference during extinction. Interaction (Time × Group), F(17, 255) = 1.087, p = 0.3666; Group, F(1, 15) = 13.64, **p = 0.0022, Sidak’s multiple comparisons test. WT, n = 7; *Nbea*+/−, n = 10. Extinction recall: There was a significant difference in freezing level during extinction recall. WT (26.51 ± 3.072, n = 7) vs *Nbea*+/− (41.82 ± 3.762, n = 10), *p = 0.0101, unpaired t-test, two-tailed. Spontaneous recovery: There was a significant difference in freezing level during extinction recall. WT (25.86 ± 2.945, n = 7) vs *Nbea*+/− (39.07 ± 4.416, n = 10), *p = 0.0395, unpaired t-test, two-tailed. (**C**) Schematic representation of the experimental protocol. (**D**) Fear conditioning: There was no significant difference in freezing level during fear conditioning between WT (n = 10) and *Nbea*+/− (n = 9). Two-way ANOVA, F(4, 68) = 0.6439, p = 0.6330. Extinction: There was a group difference during extinction. Interaction (Time × Group), F(17, 289) = 0.9358, p = 0.5320; Group, F(1, 17) = 12.69, **p = 0.0024, Sidak’s multiple comparisons test. WT, n = 7; *Nbea*+/−, n = 10. Extinction recall: There was a significant difference in freezing level during extinction recall. WT (38.53 ± 3.568, n = 10) vs *Nbea*+/− (54.9 ± 3.924, n = 9), **p = 0.0066, unpaired t-test, two-tailed.

Due to the limitation of poor contextual extinction of surgerized mice and the variable infection rate and efficiency of the knockdown virus, we decided to generate *Nbea* knockout mice. This approach allowed us to perform extensive behavior analysis to further validate the effect of *Nbea* deficiency in fear reconsolidation or extinction including within-session extinction. We generated *Nbea* null mutant mice utilizing TALEN technology. By targeting exon 22, we obtained a 17-bp out-of-frame deletion mutant as shown in Supple Fig. S1A. The 17-bp deletion created a premature stop codon immediately after the truncation in exon 22. Genomic deletion was validated by the analysis of tail DNA with PCR. The deletion created a new *Bsl*I enzyme target sequence in the genomic DNA of the mutant allele. Mice with a homozygous mutation were embryonic lethal; therefore, only heterozygotes (indicated as *Nbea*+/− from here on) were used for assays and behavior tests. First, we confirmed the genomic deletion with the *Bsl*I restriction enzyme, which generates two bands (294 bp and 90 bp) in the mutant allele (Supple Fig. S1B). We also confirmed inhibition of the NBEA protein in *Nbea*+/− using Western blot analysis (Supple Fig. S1C; t(6) = 5.149, *p = 0.0021, unpaired t-test, two-tailed). For the behavior analysis, we first recorded the body weight of these mice since gene-trap-mediated null mutant mice (GH240) have previously shown dwarfism, but there were no significant differences in body weight at 10 weeks (Supple Fig. S1D; t(19) = 1.949, p = 0.0663, unpaired t-test, two-tailed) or 16 weeks (Supple Fig. S1D; t(12) = 1.468, p = 0.1678, unpaired t-test, two-tailed). To detect any phenotypic differences in general behaviors such as locomotor activity and anxiety-like behavior, the open field test was performed. *Nbea*^+/−^ were hyperactive during the initial 5 min of the test, but their locomotor activity returned to normal levels for the rest of the exploration period (Supple Fig. S1E; F(5, 215) = 2.719, *p = 0.0210, two-way ANOVA). We also analyzed the time spent in the center zone of the arena as a measure of the anxiety level but found no difference between WT and *Nbea*^+/−^ (Supple Fig. S1F; t(43) = 1.31, p = 0.1970, unpaired t-test, two-tailed). We examined fear conditioning and extinction in these mutant mice (Fig. 5A). Since the mutant was generated in the C57BL/6J background, we increased the intensity of shocks (0.7 mA, 1 s) to achieve a certain level of recall memory for further investigation of the extinction process. Using this protocol, we did not detect any differences in acquisition or 24-h context memory recall (Fig. 5B; F(4, 60) = 2.19, p = 0.0809, two-way ANOVA). Since *Nbea*^+/−^ did not show any impairments in fear acquisition or contextual fear memory at the behavioral level, and NBEA expression was increased immediately after retrieval at the molecular level, we first tested the *Nbea*^+/−^ on the retrieval-extinction protocol. *Nbea*^+/−^ showed no difference in retrieval (Fig. 5B; t(15) = 1.824, p = 0.0881, unpaired t-test, two-tailed) and showed normal extinction learning (Fig. 5B; Time × Group interaction: F (17, 255) = 1.087, p = 0.3666, two-way ANOVA). However, there were differences in the freezing level between genotypes (Fig. 5B; Group: F(1, 15) = 13.64, *p = 0.0022, two-way ANOVA), suggesting that extinction was attenuated in *Nbea*^+/−^. The 24 h-extinction memory (t(15) = 2.944, *p = 0.0101, unpaired t-test, two-tailed) and spontaneous recall memory (t(15) = 2.255, *p = 0.0395, unpaired t-test, two-tailed) were significantly greater in *Nbea*^+/−^ than in WT (Fig. 5B), suggesting that *Nbea* is critical for fear extinction. To validate the role of *Nbea* in fear extinction, we performed an extinction protocol without retrieval (Fig. 5C). As shown previously (Fig. 5B), we did not detect any differences in fear conditioning between the two genotypes (Fig. 5D; F(4, 68) = 0.6439, p = 0.6330). However, *Nbea*^+/−^ showed normal extinction learning (Fig. 5D; Time × Group interaction: F(17, 289) = 0.9358, p = 0.5320) but attenuated extinction of the memory, indicated by higher freezing levels in *Nbea*^+/−^ than in WT (Fig. 5D; Group: F(1, 17) = 12.69, **p = 0.0024). The impaired extinction in *Nbea*^+/−^ was confirmed by significant differences in extinction recall between WT and *Nbea*^+/−^ (Fig. 5D; t(17) = 3.094, **p = 0.0066, unpaired t-test, two-tailed). Altogether, *Nbea*^+/−^ showed some tendency to increase the freezing level in retrieval, but the impairments in extinction processes were significant compared to WT, suggesting that NBEA may play a critical role extinction processes.

## Discussion

Here, we performed an unbiased proteomics analysis to identify molecules involved in fear memory retrieval. We found that NBEA is rapidly and transiently upregulated in the dorsal hippocampus after fear memory retrieval. We also found that NBEA is enriched at the synapse, and its expression is regulated by the mTOR signaling pathway. Suppression of NBEA expression, either in the whole body or locally in the hippocampal CA1 region, impaired extinction, suggesting that NBEA may play a role in regulating fear extinction processes.

Exposure therapy is a behavior therapy used to treat anxiety disorders such as PTSD^29^. To overcome their distress, patients are exposed to a feared object or context without any danger present. Contextual information and the memory process is important for this form of therapy^30^; therefore, we utilized a contextual fear conditioning protocol (Fig. 1A). Since the dorsal hippocampus is an integration center for context-dependent fear memory processing^31^, we focused on identifying molecules within this structure in this study. The retrieval-mediated labile state of memories has been suggested to be an important factor in the permanent erasure of memory^8,32^, but the specific molecules involved in these processes are not known. Through proteomic analysis, we identified NBEA as one of the most prominently upregulated proteins in the hippocampus after fear memory retrieval. Given that NBEA is a trafficking molecule that targets neurotransmitter receptors to synapses, and protein sampling was performed 5 min after a 5-min retrieval session, we questioned whether an increase in the expression of NBEA after fear retrieval could be detected at the synapse. Local synaptic protein synthesis is known to be very rapid (within 5 min)^25,33,34^. Using synaptosomal fractionization, we clearly observed an increase in NBEA expression at 5 min after retrieval in the dorsal hippocampus (Fig. 1C,D). Thereafter, we only focused on NBEA expression at the synaptic level when examining the molecular dynamics of NBEA after retrieval. The time course of NBEA expression after retrieval (Fig. 2B,C) was similar to that of other proteins regulated by local translation^25^. The mTOR signaling pathway, also called the local protein synthesis pathway, is the main pathway that regulates the rapid induction of protein synthesis from existing mRNA at the synapse^35^. Rapamycin clearly inhibited the rapid induction of NBEA expression after retrieval, suggesting that retrieval indeed activates the new synthesis of NBEA at the synapse (Fig. 3). Therefore, the dynamic changes in NBEA expression appear to be necessary for the state-dependent modulation of fear memories, especially after fear memory retrieval. Interestingly, we did not observe any induction of NBEA after neutral-context retrieval, indicating that rapid NBEA protein induction at the synapse is closely related to fear retrieval (Fig. 1G,H). However, different sets of experiments may be necessary to test whether the relationship between NBEA expression and fear retrieval is generalizable to other types of retrieval learning that are not fear related.

Previous studies have shown that the mTOR signaling pathway is critical for the consolidation of fear memory reconsolidation^27,28,36^ and extinction^37^. In these studies, treatment with the mTOR inhibitor, rapamycin, immediately before or after retrieval impaired memory reconsolidation^27^, which is not consistent with our data. Although NBEA expression was regulated by mTOR, we found either increased fear memory or impaired extinction in both *Nbea* knockdown (Fig. 4) and *Nbea*^+/−^ mice (Fig. 5). However, several studies support our data as well. For example, ketamine treatment, which is known to activate mTOR, accelerated the extinction process^10^. Another protein synthesis inhibitor, anisomycin, impaired extinction^38^. Therefore, specific mTOR downstream pathways may play different roles in memory stabilization and destabilization. In support of this idea, a study reported that two distinct molecular mechanisms are involved in the destabilization and restabilization of reactivated spatial memory^39^. In other words, inhibition of all proteins regulated by mTOR may mask the effects of a sub-group of proteins, including NBEA, involved in memory destabilization.

The mechanism by which the rapid induction of NBEA expression affects extinction is not clear since retrieval and extinction are known to be two distinct memory processes^40^. However, several studies have suggested a molecular link between retrieval and extinction^4,41^. NBEA is a regulator of ionotropic receptor trafficking to synapses^13^, and ionotropic glutamate receptor plasticity is required for memory consolidation and reconsolidation^42^. The learning, consolidation and reconsolidation of long-term memory require stable postsynaptic potentiation that is modulated by an increase in postsynaptic α-amino-3-hydroxy-5-methyl-4-isoxazolepropionic acid (AMPA) receptors (AMPARs)^43^. AMPARs are tetrameric glutamate receptors that have different properties depending on their subunit composition^44^. The majority of synaptic AMPARs are calcium-impermeable AMPARs (CI-AMPARs) that contain the GluA2 subunit and are stable at the synapse due to the interaction of the GluA2 subunits with synaptic molecules^45^. Moreover, most basal synaptic transmission is regulated through CI-AMPARs. On the other hand, GluA2-lacking AMPARs, calcium-permeable AMPARs (CP-AMPARs) are unstable at the synapse but involved in acute synaptic potentiation^45^. A recent study revealed the physiological roles of CI-AMPARs and CP-AMPARs in transforming a consolidated memory into an unstable memory and subsequently guiding extinction or reconsolidation^4^. In their results, memory retrieval caused a transient replacement of CI-AMPARs with CP-AMPARs. Furthermore, blocking CI-AMPAR endocytosis prevented the insertion of CP-AMPARs, thereby preventing the transformation of a consolidated memory to a labile state and leading to increased fear memory. Here, most intriguingly of all, the temporal profile of CP-AMPARs was very similar to that of NBEA expression after retrieval. Since we found that mice with *Nbea* KD showed enhanced fear memory (Fig. 4F), and the temporal patterns of NBEA and CP-AMPARs after retrieval are very similar (Fig. 2B,C), NBEA is speculated to regulate CP-AMPAR trafficking to the synapse. In support of this, NBEA interacts with protein kinase A (PKA)^11^, which phosphorylates GluA1-S845. GluA1-S845 phosphorylation enhances the mean channel open probability and promotes synaptic trafficking of GluA1-containing AMPARs (CP-AMPARs), especially to extrasynaptic sites. Therefore, in extinction, NBEA may alter the subunit composition of perisynaptic AMPARs by providing stability to GluA1 homomers, making NBEA necessary for the maintenance of CP-AMPARs on the surface, which is required for the extinction process^46^. In support of this idea, we found that NBEA interacts with PKA and GluA1 but not with GluA2 (Supple Fig. S3). Interestingly, AKAP150, which is one of major forms of PKA binding protein regulating synaptic transmission in the hippocampus, did not show dynamic changes in expression after retrieval, suggesting that NBEA might be an activity-dependent regulator that modulates PKA and AMPA receptor phosphorylation associated with contextual fear retrieval (Fig. 2D,E). Further analysis of the biochemical and electrophysiological role of NBEA in this process is required.

We generated *Nbea* null mutant mice, but the mutation was embryonic lethal. Therefore, we only used heterozygotes for our behavior assays. *Nbea* mutant mice have been generated in two different labs using gene-trap methods. Both *Nbea* knockout mouse lines showed perinatal lethality^20,47^. In the case of the GB140 line, several behavioral analyses have been performed. *Nbea*^+/−^ showed enhanced conditioned fear memory and impaired spatial learning and memory as well as enhanced long-term potentiation in the CA1 area of the hippocampus^19^. However, we did not see enhanced conditioned fear memory compared to that in WT littermates. The group cited above used 24-month-old female mice for the tests, whereas we used 10–16-week-old males. In addition, dwarfism of *Nbea*^+/−^ was reported^18^, but we did not see any differences in body weight between *Nbea*^+/−^ and WT (Supple Fig. S2D). However, we did observe hyperactivity in the open field test, which was not shown in the previous GB140 line. Therefore, we believe that the differences in the phenotypes between GB140 and this newly generated *Nbea*^+/−^ mouse line are due to several factors, such as the method used to generate the knockout mouse line (gene trapping for GB140 vs TALENs for our study), the dwarfism, the gender and age of the test groups.

Taken together, this study demonstrated that NBEA plays a role in either the induction of memory lability or the physiological process of memory extinction. Our research showed activity-dependent activation of NBEA at the synapse after retrieval, and impaired extinction by the dysregulation of NBEA in either the hippocampal CA1 or the whole body. Therefore, we suggest NBEA as a novel target for research on how it may contribute to PTSD extinction deficits or the efficacy of exposure therapy. Furthermore, regarding the possible role of *Nbea* as an autism molecule, this study may also facilitate research to identify potential pathways connecting PTSD and ASD^48^.

## Materials and Methods

### Animals

Adult male wild-type (WT) mice (10–16 weeks of age) of B6x129 F1 or B6 background were used for experiments. F1 mice were obtained by mating the parental strains C57BL/6J (N20-23) and 129s4/svJ (N20-23). B6x129 F1 mice were used because we used this strain in most of our previous fear and extinction studies^49,50^, and these mice were used for proteomics, western blot, immunoprecipitation and *shNbea* knockdown experiments. *Nbea*^+/−^ mice were generated on the B6 background and were also included in the western blot analysis shown in Fig. 1E,F,G. All mice were housed with free access to food and water under controlled temperature and light conditions (23 °C, 12-h light: 12-h dark cycle). Experiments were performed during the light phase. All experimental procedures and animal care were performed in accordance with the guidelines approved by the Institutional Animal Care and Use Committee (IACUC) of the Institute for Basic Science (IBS), Korea. All methods were performed in accordance with the relevant guidelines and regulations.

### Preparation of protein extract for proteomics

Five minutes after contextual fear retrieval, the hippocampus was dissected and washed with ice-cold 1X phosphate-buffered saline (PBS) to reduce blood contamination, followed by lysis in 500 µl of lysis buffer (6 M urea, 50 mM Tris-Cl, pH 8.3, 5 mM EDTA, 0.05% SDS, 1x protease inhibitor cocktail, and 1x phosphatase inhibitor cocktail) by sonication at 4 °C for 1 min with an intermittent cooling period. After centrifugation at 12,000 rpm for 15 min at 4 °C, the supernatants were collected. The concentration of total protein was determined by Bradford assay. The tissue lysates were stored at −80 °C until use.

### Protein digestion for mass spectrometry

A total of ten samples from two groups (5 mice per group, 40 µg lysates per sample) were adjusted to the same protein concentration, reduced with 10 mM dithiothreitol (DTT) for 30 min at 37 °C and then alkylated with 40 mM iodoacetamide for 1 h in the dark at 25 °C. After the samples were diluted 10-fold with 50 mM NH_4_HCO_3_, trypsin was added at a ratio of 1:40 (w:w), followed by incubation overnight at 37 °C. Two peptide mixtures were applied to a C18 cartridge. Purified peptide samples were dried and stored at 4 °C until liquid chromatography-tandem mass spectrometry (LC-MS/MS) analysis.

### TMT-labeling proteomic analysis

The peptide mixture of the two groups (control and retrieval group) was used for tandem mass tag (TMT) reagent labeling according to the manufacturer’s protocols (Thermo Fisher Scientific, Waltham, MA, USA). Each sample was then labeled with one of two different TMT reagents (TMT126 for control and TMT129 for retrieval) according to the manufacturer’s protocols. After the labeling, the TMT-labeled peptide pool was cleaned with a C18 cartridge. Then, an OFFGEL electrophoresis was performed according to the manufacturer’s protocols (Agilent, Santa Clara, CA, USA). Desalted and dehydrated samples were reconstituted in OFFGEL solution. Isoelectric focusing was performed on an immobilized pH gradient (IPG) dry strip (13 cm, pH 3–10, linear; GE Healthcare, Chicago, IL, USA) using a 12-well frame for 20 kVh with a maximum current of 50 μA and power of 200 mW. The collected fractions were desalted using C18 spin columns (Thermo Fisher Scientific, Waltham, MA, USA), then evaporated by a speed vacuum and stored at −20 °C. LC-MS/MS analysis was performed using an LTQ Orbitrap XL (San Jose, CA, USA). The spray voltage was set to 1.9 kV, and the temperature of the heated capillary was set to 25 °C. The sample was subjected to reverse-phase chromatography using an Eksigent LC system (AB Sciex, Framingham, MA, USA). The mobile phases consisted of H_2_O (A) and acetonitrile (ACN) (B), where both phases contained 0.1% v/v formic acid. The flow rate was maintained at 300 nl/min. The peptides were eluted from the analytical column (C18) by a linear gradient running from 10% to 35% acetonitrile over 95 min to 80% B over the next 20 min and to 90% B over the final 15 min, followed by direct spray into the LTQ mass spectrometer. Mass spectra were acquired in the positive mode with an m/z window of 300–2,000, and a maximum of 3 precursors were selected for higher energy collisional dissociation (HCD) analysis in the Orbitrap (isolation width: 2; min. signal required: 1000; normalized collision energy: 45%) with dynamic exclusion. SEQUEST (XCorr Only) (ThermoFinnigan, San Jose, CA; version v.1.3.0.339) searches were performed against tryptic peptides, allowing for two missed cleavages with 25 ppm (monoisotopic) precursor mass tolerance and 25 ppm (monoisotopic) fragment mass tolerance detected in the Orbitrap. The searches were conducted against a mouse database (IPI ver. 3.87) with the decoy option enabled, and the iodoacetamide derivative of cysteine and TMT multiplexed quantitation chemistry of lysine and the N-terminus were specified in SEQUEST as fixed modifications. Methionine oxidation (+15.9949) was specified in SEQUEST as a variable modification. Scaffold (version Scaffold_4.0.5, Proteome Software Inc., Portland, OR) was used to validate MS/MS-based peptide and protein identifications. Peptide identifications were accepted if they could be established at a probability greater than 0.9 by the peptide prophet algorithm. Protein identifications were accepted if they could be established at a probability greater than 0.9 and contained at least 2 identified unique peptides. Protein probabilities were assigned by the protein prophet algorithm. Proteins that contained similar peptides and could not be differentiated based on MS/MS analysis alone were grouped to satisfy the principles of parsimony. For the TMT quantification, the LIBRA (Trans-proteome pipeline, version 4.2.1) program was used to quantitate the label-based quantitation peptide and protein identifications. A fold change greater than 1.2 or less than 0.8 was considered statistically significant^51,52^.

### Preparation of the synaptosome and immunoblotting analysis

Synaptosome purification has been described previously^26^. For the validation of NBEA expression after retrieval, whole hippocampal tissue was used. For the rest of the study, the dorsal hippocampus was dissected and homogenized in ice-cold lysis buffer using a Teflon-glass tube. For immunoblotting analysis, antibodies targeting the following proteins were used. Primary antibodies against the following antigens were used: synapsin I (1:1,000, Abcam, Cambridge, UK), postsynaptic density 95 (PSD95; 1:1,000, Abcam, Cambridge, UK), histone H1 (1:1,000, Abcam, Cambridge, UK), NBEA (K-20) (1:1,000, Santa Cruz Biotech., Dallas, TX, USA), A kinase anchor protein 150 (AKAP150; 1:1,000, Santa Cruz Biotech, Dallas, TX, USA) and glyceraldehyde 3-phosphate dehydrogenase (GAPDH; 1:5,000, Cell signaling, Danvers, MA, USA). The secondary antibodies were horseradish peroxidase (HRP)-conjugated anti-mouse (1:5,000) or anti-rabbit (1:10,000) antibodies (PerkinElmer Life Sciences, Norwalk, CT, USA). Signal detection was performed using the LAS 2000 system (LAS-2000, Fuji, Tokyo, Japan) with enhanced chemiluminescence (Western-CDP star, PerkinElmer Life Sciences, Norwalk, CT, USA). Densitometric analysis of immunoreactivity for each protein was conducted using NIH ImageJ software.

### Real-time PCR

Total RNA or synaptosomal RNA was isolated with GeneAll Hybrid-R, and cDNA was synthesized with a cDNA synthesis kit (Superscript Vilo, Invitrogen, Waltham, MA, USA). Then, PCR amplification was performed using 5X Qarta-probe qPCR mix (Qarta bio, Carson, CA, USA). Real-time quantitative RT-PCR was carried out using TaqMan Gene Expression assays and Applied Biosystems (ABI) Step One Plus (Applied Biosystems, Waltham, MA, USA). Primer sequences for *Nbea* were 5′-CCATCTATCCCTCATCCAAGTTT-3′ (sense), 5′-CCATGGACTCCTTGCCA AGCTGAT-3′ (probe), and 5′-CCTTCTCCTTTACAGTGTCTGG -3′ (antisense); the primer sequences for the internal control, beta-2 microglobulin, were 5′-GGTCTTTCTGGTGCTTGTCT-3′ (sense), 5′-CAAGTATACTCACGCCACCCACCG-3′ (probe), and 5′-TATGTTCGGCTTCCC ATTCTC-3′ (antisense). The PCR conditions were as follows: initial incubation at 50 °C for 2 min and denaturation at 95 °C for 10 min, followed by 40 cycles of 95 °C for 15 s and 60 °C for 1 min.

### Drug delivery

For microinjection, cannulas (PlasticsOne, Roanoke, VA, USA) were bilaterally implanted with a 26-gauge guide cannula in the CA1 of the dorsal hippocampus (anterior-posterior (AP) −1.9 mm, lateral (L) +1.2 mm or −1.2 mm, and dorsal-ventral (DV) 1.5 mm from bregma) using a stereotaxic apparatus (Kopf Instruments, Tujunga, CA, USA) under 2% Avertin anesthesia. A 33-gauge dummy cannula was inserted into each guide cannula to prevent clogging. The injection cannulas were cut to extend 0.2 mm beyond the guide cannulas. After 7 days of recovery, mice received bilateral 5-min infusions of DMSO (1 μl each) or rapamycin (1 μl each, 50 μM in DMSO) into the CA1 of the dorsal hippocampus, and the injection cannula remained in place for an additional 5 min to ensure diffusion away from the injector tip. Behavioral experiments were performed 30 min after the drug infusion. Thirty minutes after retrieval, the dorsal hippocampal tissue was isolated and western blot analysis was performed as described above. The control DMSO or rapamycin-only group was sacrificed 1 hour after drug infusion to match with the sampling time of the retrieval DMSO or rapamycin group.

### Primary hippocampal neuron culture

Primary cultures of murine hippocampal neurons were done as previously described^53^, with minor modifications. A papain dissociation system (Worthington biochemical cooperation, Lakewood, NJ, USA) was used to dissociate murine hippocampal neurons. After mechanical trituration and centrifugation, the resulting cell pellet was resuspended in culture medium containing Neurobasal-A medium (Gibco, Waltham, MA, USA), 2% B27 serum (Gibco, Waltham, MA, USA), and 1x penicillin/streptomycin (Gibco, Waltham, MA, USA). Afterward, cells were plated at a high density of 5 × 10^5^ cells/ml in 24-well plates (BD Bioscience, San Jose, CA, USA) coated with 1 mg/ml poly-D-lysine hydrobromide (Sigma-Aldrich, St. Louis, MO, USA). Hippocampal neurons were cultivated at 37 °C and 5% CO_2_ in a humidified incubator. After the cells were allowed to adhere for 3 h, more culture medium was added, and the cells were maintained for 10–14 days in culture before use. On day 5, 1 μl of high-titer adeno-associated virus (AAV; 1 × 10^12^ genome copy/ml) was added. The culture was maintained for an additional 5 days and then harvested for the protein assay.

### AAV vector production

Separate hairpin RNA (shRNA) constructs were designed to target *Nbea* mRNA. Cloning was performed as previously described^54^. Briefly, DNA oligomers were synthesized and cloned into a modified pAAV-MCS vector, pAAV-shRNA (provided by Dr. Ralph J. DiLeone, Yale University School of Medicine). The loop sequence (CTTCCTGTCA) was inserted between the antisense (GAAGTCATGCAATTGACTGTGTGA) and sense (TCACACAGTCAATTGCATGACTTC) sequences. For control, the following scrambled shRNA sequence was used: GATTATACACCGACAATTCCGTGT (antisense) and ACACGGAATTGTCGGTGTATAATC (sense). The insertion was confirmed by sequencing. High-titer AAV virus (1 × 10^12^ genome copy/ml) was packaged with serotype DJ at KIST virus facility (http://virus.kist.re.kr).

### Tissue preparation and immunohistochemistry

Mice were deeply anesthetized with Avertin (20 μl/g, intraperitoneally, i.p.) and transcardially perfused with PBS (pH 7.4) followed by a fixative, 4% paraformaldehyde in 0.01 M PBS (pH 7.4) at room temperature. Brains were removed from the skull and post-fixed overnight in the same solution at 4 °C. Using a vibrating microtome (Leica Microsystems, Wetzlar, Germany), we cut the brain containing hippocampal tissue into 50-μm-thick sections. Immunohistochemistry was done as previously described (Lee *et al*., 2009). Antibodies targeting the following proteins were used: NBEA (1:1000; Santa Cruz Biotech., Dallas, TX, USA) and NeuN (1:500; Millipore, Burlington, MA, USA). The Cy5-conjugated donkey anti-rabbit IgG antibody (1:300; Jackson ImmunoResearch, West Grove, PA, USA) and DyLight 405-conjugated donkey anti-mouse IgG antibody (1:300; Jackson ImmunoResearch, West Grove, PA, USA) were used as secondary antibodies. After the sections were washed with PBS, they were mounted in Vectashield (Vector Laboratories, Burlingame, CA, USA) and examined under a confocal laser-scanning microscope (Nikon, Shinagawa-Ku, Tokyo, Japan).

### Open field test

The open field test was conducted as previously reported^55^. Briefly, each mouse was placed in a white acrylic chamber (40 × 40 × 40 cm) for 30 min to measure its locomotor activity. At the beginning of the test, the mouse was placed in the corner of the arena. To examine the anxiety level of the mouse, the exploration time in the center (20 × 20 cm) during the first 5 min was analyzed. Spontaneous movement was video recorded and automatically analyzed with EthoVision XT software, version 9 (Noldus, Wageningen, Netherlands).

### Contextual fear conditioning, retrieval and extinction

Contextual fear conditioning was performed as previously described^56^, with minor modifications. All animals were handled for three days for 10 min each. As shown in Fig. 1, for the fear conditioning of mice used in the proteomic analysis and validation of the proteomic results, mice from both control and retrieval groups were placed in the conditioning chamber and received a foot shock at 180 s (0.5 mA, 1 s) through the rod floor. Then, mice were returned to their home cage 60 s after the end of the last shock. Twenty-four hours after training, the control group was placed in a novel context for 5 min. The retrieval group was placed in the same chamber in which conditioning occurred for 5 min. For time course studies and behavior analysis, two foot shocks were delivered at 180 s and 240 s (0.5 mA, 1 s for F1 mice for proteomic study, time course biochemistry and knockdown experiments, 0.7 mA, 1 s for B6 mice for knockout experiments). For the no-retrieval group, mice received foot shocks for conditioning, just as the retrieval group did, but were not placed in the same context as retrieval to examine baseline expression of NBEA 24 hrs after conditioning and before retrieval. For the retrieval-extinction experiment, 30 min after retrieval, mice were again placed in the same chamber for another 18 min, and 24 h later, the mice were placed in the same chamber for 5 min to measure extinction memory. For the extinction experiment, 24 h later, without a 5-min retrieval session, mice were directly exposed to the same context for 18 min, and extinction memory was observed for 5 min in the same context 24 h later. For the spontaneous recovery test, the memory was tested in the same chamber 2 weeks later for 5 min. Baseline activity, exploration and freezing were assessed automatically using EthoVision software (Noldus Information Technology, Wageningen, Nederland).

### Statistical analysis

Data were analyzed using GraphPad Prism 7.03 (GraphPad Software Inc., La Jolla, CA, USA). Comparisons between two groups were determined by Student’s *t*-test. To analyze the time course expression of NBEA or AKAP150 after retrieval and conditioning, one-way ANOVA with post hoc Dunnett’s multiple comparisons test was used. To analyze the NBEA expression results shown in Fig. 1D,G, one-way ANOVA with post hoc Tukey’s multiple comparisons test was used. To analyze locomotor activity in the open field test and freezing in fear conditioning and extinction, two*-*way repeated measures ANOVA with post hoc Sidak’s multiple comparisons test was performed. All values indicated are means ± SEM, and the statistical significance is represented with asterisks at p values of <0.05 (*), <0.01 (**), <0.001 (***), and <0.0001 (****).

## Electronic supplementary material

Supplementary Material
