# Supplementary material for: The Possible Role of Neurobeachin in Extinction of Contextual Fear Memory
Source: Sci Rep. 2018 Sep 13;8:13752. doi: 10.1038/s41598-018-30589-1 (PMC6137154; doi:10.1038/s41598-018-30589-1)
Supplement: Supplementary file 1 — Supplementary Material [file 41598_2018_30589_MOESM1_ESM.pdf]

The Possible Role of Neurobeachin in Extinction of Contextual Fear Memory

Boyoung Lee1, Eunyoung Bang1, Won Suk Yang3, Afshin Paydar1, Goeun Ha5, Sujin Kim1,2, Jong-Hyun Kim1,3,4, Taesup Cho1, Seung Eun Lee3, Sukchan Lee1, Myoung-Goo Kang1, Eunji Cheong5, Key-Sun Kim3, Hee-Sup Shin1,2

Affiliations

1 Center for Cognition and Sociality, Institute for Basic Science, Daejeon 34047, Republic of Korea;

2 Basic Science, IBS School, University of Science and Technology, Daejeon 34113, Republic of Korea;

3 Brain Science Institute, Korea Institute of Science and Technology, Seoul 02792, Republic of Korea;

4 Laboratory of Cell Death and Human Diseases, Department of Life Sciences, School of Life Sciences, Korea University, Seoul 02841, Republic of Korea;

5 Department of Biotechnology, College of Life Science and Biotechnology, Yonsei University, Seoul 03722, Republic of Korea.

***Corresponding authors**

H.-S. Shin, MD, PhD, Center for Cognition and Sociality, Institute for Basic Science (IBS), Korea Advanced Institute of Science and Technology (KAIST), Bd E18, 291 Daehak-ro, Yuseong-gu, Daejeon 34047, Republic of Korea. shin@ibs.re.kr

Supplemental Information

Materials and Methods

Tissue preparation and immunohistochemistry

Immunohistochemistry was performed as previously described (Lee et al., 2009). Antibodies targeting the following proteins were used: γ-aminobutyric acid (GABA; 1:1000, Millipore, Burlington, MA, USA), parvalbumin (1: 1000, Millipore, Burlington, MA, USA), and NBEA (1:1000; Santa Cruz Biotech., Dallas, TX, USA). The Cy3-conjugated donkey anti-rabbit IgG antibody (1:300; Jackson ImmunoResearch, West Grove, PA, USA) and Cy5-conjugated donkey anti-mouse IgG antibody (1:300; Jackson ImmunoResearch, West Grove, PA, USA) were used as secondary antibodies. The sections were counterstained with 4',6-diamidino-2-phenylindole (DAPI; 1 μg/ml; Thermo Fisher Scientific, Waltham, MA, USA). After the sections were washed with PBS, they were mounted in Vectashield (Vector Laboratories, Burlingame, CA, USA) and examined under a confocal laser-scanning microscope (Nikon, Shinagawa-Ku, Tokyo, Japan).

Generation and genotyping of the targeted mouse *Nbea* knockout mutant

Transcription activator-like effector nuclease (TALEN) vectors that targeted exon 22 of *Nbea* were designed and constructed by ToolGen (Seoul, Korea). The synthesis and microinjection of TALEN mRNAs into the cytoplasm of fertilized eggs obtained from C57BL/6J breeding females were performed as previously described (May et al., 2015). TALEN-mediated *Nbea* F0 mice were screened via the T7 endonuclease I (T7E1) assay as previously described (Sung et al., 2013). For the assay, genomic DNA was prepared from the tail and amplified using TALEN target site primers. The primers used for genotyping included mouse *Nbea* forward (5'-ZAGAGAAAGTGGAAGCCACAG-3') and mouse *Nbea* reverse (5'- TACACTCGCAGTCATGTGAGC-3'). PCR products were further digested with the *Bsl*I enzyme (New England Biolabs, Ipswich, MA, USA) to validate the deletion allele. The founder line of the *Nbea* heterozygous mouse (F0) was crossed to and maintained in the C57BL/6J background.

Immunoprecipitation

Extracts (350 μg) from the dorsal hippocampus were incubated overnight with 5 μg of either anti-NBEA (Santa Cruz Biotechnology, Heidelberg, Germany) antibody or normal rabbit IgG (Cell Signaling Technology, Danvers, MA, USA) in the presence of Protein G beads using a Protein G Immunoprecipitation kit (Sigma-Aldrich, Buchs, Switzerland), and the resulting complexes were washed, denatured and eluted according to the manufacturer's instruction. The eluted immunoprecipitate was loaded on an SDS-PAGE gel and processed for western blot. The following antibodies were used: NBEA, PKAIIα, AKAP150 (Santa Cruz Biotech., Dallas, TX, USA), GluA1, and GluA2 (Abcam, Cambridge, UK). Mouse anti-rabbit IgG (conformation specific) secondary antibody (Cell Signaling Technology, Danvers, MA, USA) was used for detection.

Reference

1. LEE B, Cao R, Choi YS, Cho HY, Rhee AD, Hah CK, Hoyt KR, Obrietan K (2009): The CREB/CRE transcriptional pathway: protection against oxidatie stress-mediated neuronal cell death. *J Neurochem.* 108: 1251-1265.

2. Sung YH, et al. Knockout mice created by TALEN. *Nat. Biotechnol.* 2013;31:23–24. doi: 10.1038/nbt.2477.

Supplemental Figure S1

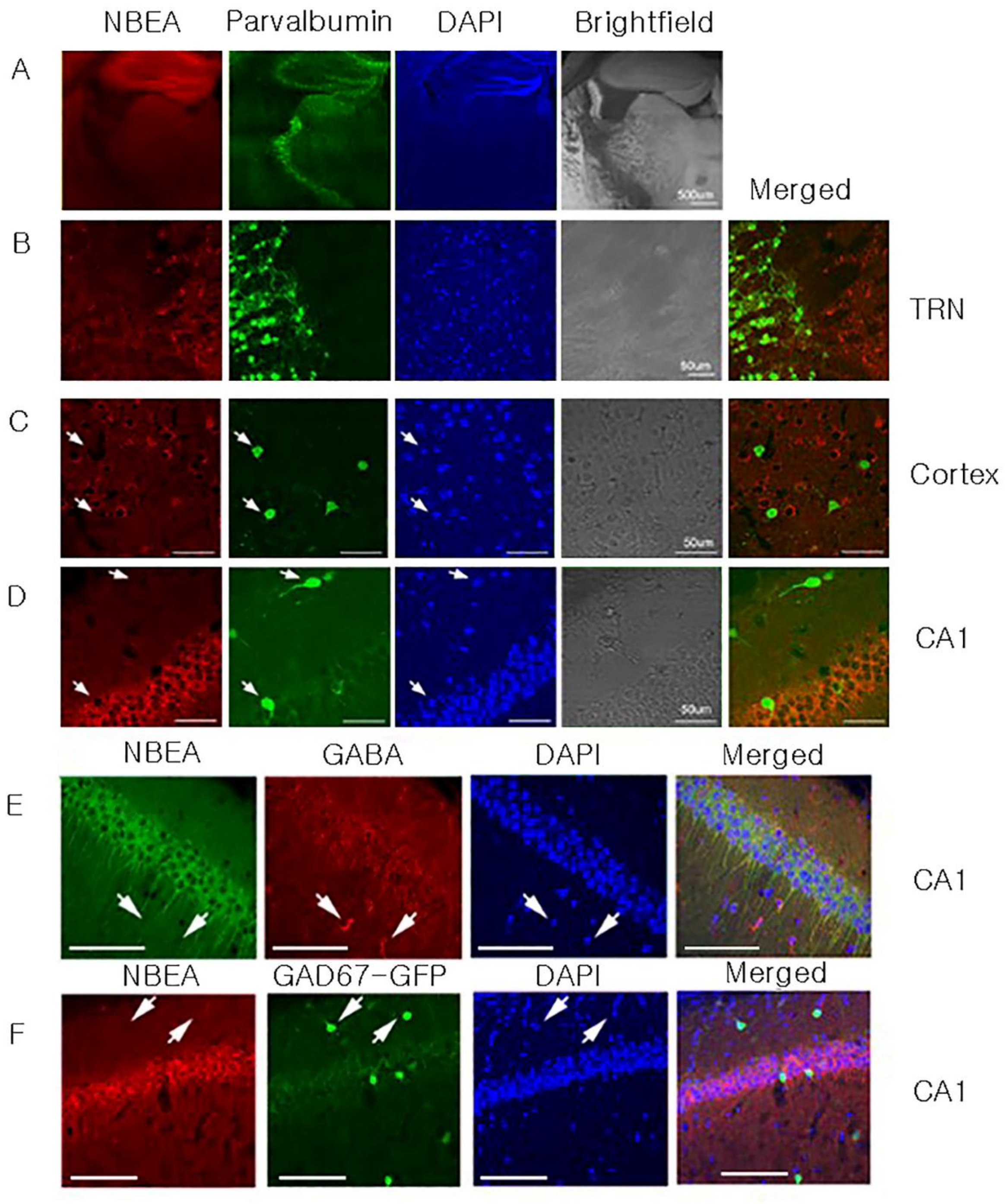

Supplemental Figure S1. NBEA expression was not seen in inhibitory neurons in the mouse brain. A, Low magnification of the mouse brain. Red: NBEA; green: parvalbumin, a maker of subpopulations of inhibitory neurons; blue: DAPI, a marker of individual cell nuclei. Brightfield image showing the

identification of the regions. Scale bar indicates 500 μm. B, TRN (thalamic reticular nucleus) and thalamus region. NBEA expression was low in the TRN region. Scale bar indicates 50 μm. C, Cortex. NBEA expression was low in parvalbumin-positive cells in the cortex. Scale bar indicates 50 μm. D, CA1 in the dorsal hippocampus. NBEA expression was low or absent in parvalbumin-positive cells in the CA1. Bar indicates 50 μm. E, CA1. Green: NBEA; red: GABA; blue: DAPI. NBEA expression was low in GABA-positive cells in the CA1. Scale bar indicates 100 μm. F, CA1. Red: NBEA; green: GFP signal from GAD67-GFP transgenic mice; blue: DAPI. NBEA expression was low in GAD67-positive cells in the CA1. Scale bar indicates 100 μm.

Supplemental Figure S2

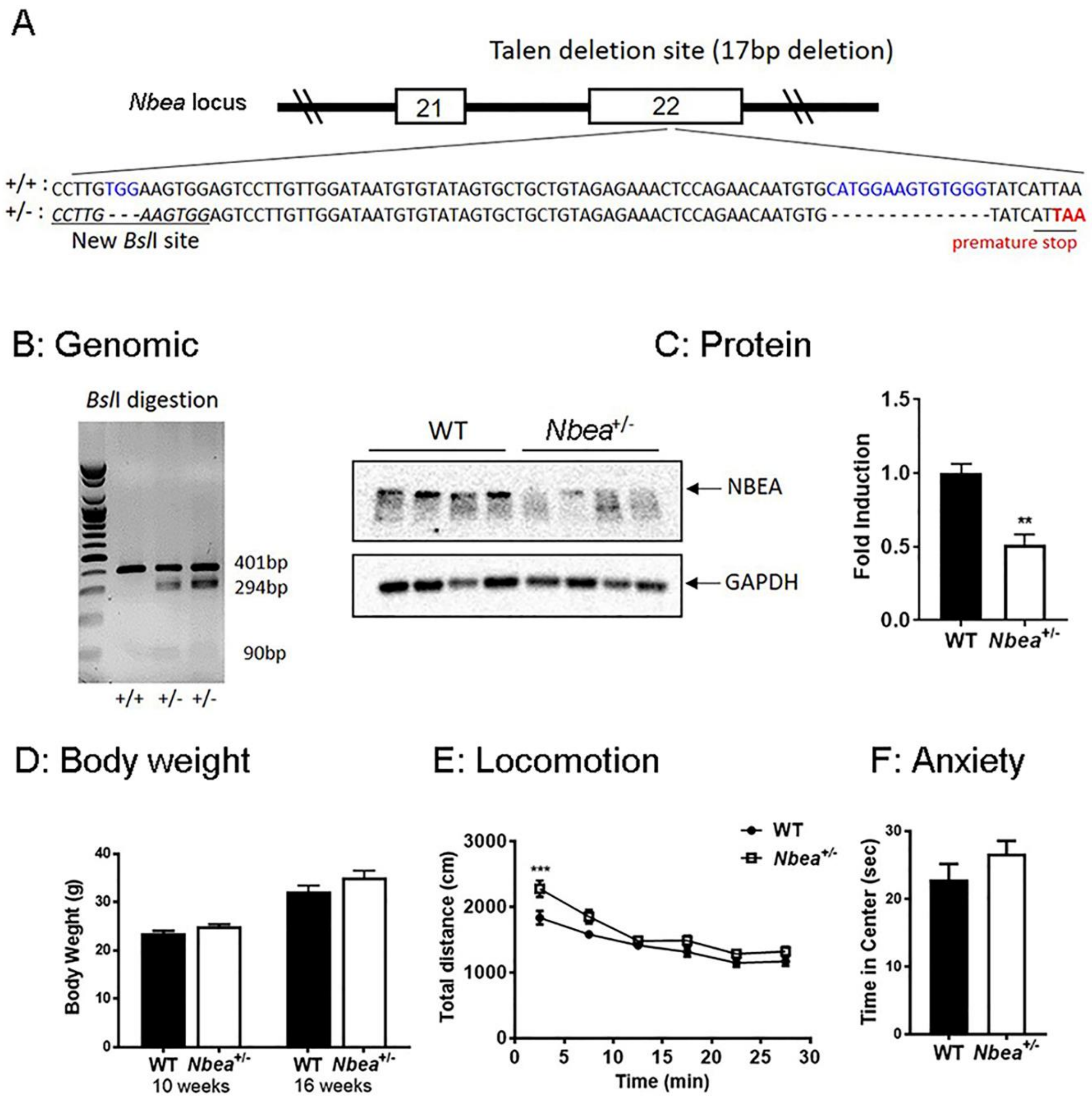

Supplemental Figure S2. Generation of *Nbea* KO mice by TALEN vectors. A, Schematic representation of the genomic structure of the mouse *Nbea* gene. The blue character sequences indicate deleted nucleotides in the WT and "–" denotes deleted nucleotides in the mutant allele. The premature stop codon is highlighted in red. B, Gel electrophoresis after BslI digestion of the PCR products of *Nbea* genomic DNA. C, Left, Western blot image of NBEA from WT and *Nbea+/-*. Right, Quantification of Western blot (right). WT (1 ± 0.06229, n=4) vs *Nbea+/-* (0.5131 ± 0.07115, n=4), **p=0.0021, unpaired t-test, two-tailed. Intensity was normalized with respect to the average intensity

of WT. D, Body weight at 10 weeks, WT (23.56 ± 0.4888, n=8) vs *Nbea+/-* (24.95 ± 0.4713, n=13), p=0.0663, unpaired t-test, two-tailed. Body weight at 16 weeks, WT (32.2 ± 1.208, n=7) vs *Nbea+/-* (35.03 ± 1.502, n=7), p=0.1678, unpaired t-test, two-tailed. E, Locomotion in the open field test. There was a significant difference in locomotor activity during the first 5 min but no difference at later time points between WT and *Nbea+/-*. F(5, 215)=2.719, *p=0.0210. Two-way ANOVA, Sidak's multiple comparisons post hoc test; 5 min, t(258)=3.954, ***p=0.0006; 10 min, t(258)=2.424, p=0.0925; 15 min, t(258)=0.6304, p=0.9891; 20 min, t(258)=1.558, p=0.5374; 25 min, t(258)=1.235, p=0.7715; 30 min, t(258)=1.358, p=0.6865. WT, n=20; *Nbea+/-*, n=25. F, Anxiety-like behavior in the open field test. There was no significant difference in anxiety level. WT (21.33 ± 2.882, n=20) vs *Nbea+/-* (25.95 ± 2.155, n=25), p=0.1970, unpaired t-test, two-tailed.

Supplemental Figure S3

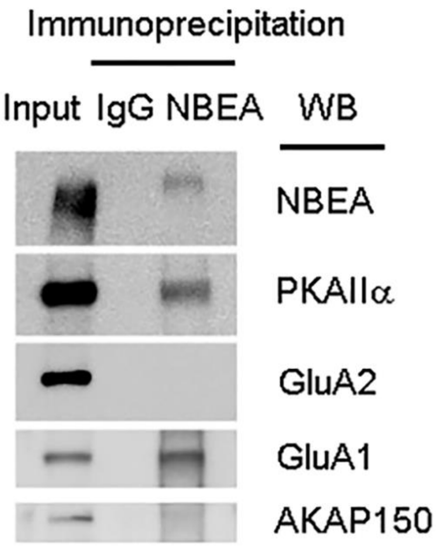

Supplemental Figure S3. NBEA interacts with PKAIIα and GluA1. PKAIIα and GluA1 were co-precipitated with NBEA, but GluA2 and AKAP150 were not in the complex. Rabbit IgG (IgG) was used as control. Input (20μg) is the protein lysates saved before immunoprecipitation. Mouse Anti-rabbit IgG (conformation specific) secondary antibody (Cell signalling technology, Danvers, MA, US A) was used for detection.

Supplemental Figure S4

Figure 1C.

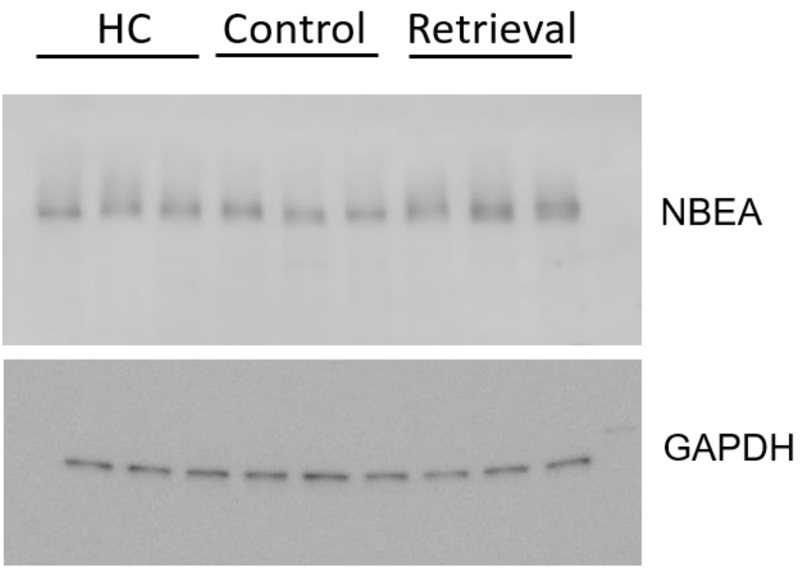

Figure 3G.

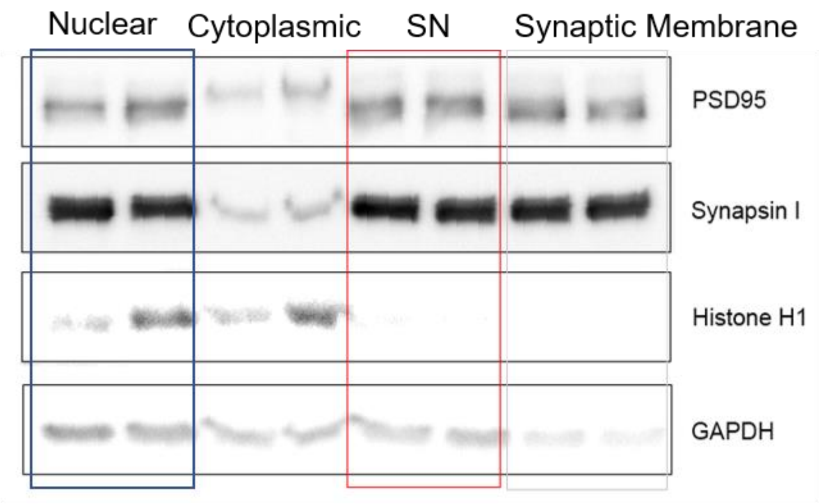

Supplemental Figure S4. Full length blots. Upper: Full length blot of Figure 1C. Lower: Full length blot for Figure 3G. Blue box indicates nuclear fraction. Red box indicates SN fraction. One of each fraction was used for representative.
